# Supplementary material for: Impact of COVID-19 Pandemic on Well-Being, Social Relationships and Academic Performance in a Sample of University Freshmen: A Propensity Score Match Evaluation Pre- and Post-Pandemic
Source: Int J Environ Res Public Health. 2023 Jul 31;20(15):6485. doi: 10.3390/ijerph20156485 (PMC10418828; doi:10.3390/ijerph20156485)
Supplement: Supplementary file 1 [file ijerph-20-06485-s001.zip › ijerph-2508202-supplementary.pdf]

International Journal of
Environmental Research and Public Health

MDPI

Supplementary Materials

Table S1. Tools description.

	Number of items	Type of response	Score	Reliability
**General Health Questionnaire (GHQ-12)**	12	4-point Likert scale from 0 (Much less than usual) to 3 (More than usual).	A score of 0 was assigned to the first two low-stress alternatives and a score of 1 was given to the two high-stress alternatives. The total score ranges from 0 to 12 (cut-off >3).	Cronbach's alpha of 0.81
**University Stress Scale (USS)**	21	4-point Likert scale from 0 (Not at all) to 3 (Constantly).	The total score ranges from 0 to 63 (cut-off ≥ 13).	Cronbach's alpha of .83
**University Connectedness Scale (UCS)**	18	7-point Likert scale, from 1 (Not at all) to 7 (All the time).	The total score ranges from 18 to 126 (no cut-off).	Cronbach's alpha of .88
**Brief COPE Inventory**	28	4-point Likert scale from 1 (I haven't been doing this at all) to 4 (I've been doing this a lot).	14 sub-scales. Each sub-scale score ranges from 2 to 8 (no cut-off).	Cronbach's alpha values ranging from .50 to .90
**Modified version of World Health Organization-ASSIST v3.0**	8 questions about the use of 10 types of substances (tobacco, alcohol, marijuana, cocaine, stimulants, sedatives, inhalants, hallucinogens, opioids, and other drugs).	The 8 questions address: the frequency of lifetime use, and in the past three months; feelings of compulsion; drug-related problems; inability to perform expected tasks; concern by family/ friends; unsuccessful attempts to stop or reduce use; injection use.	Each response corresponds to a score, ranging from 0 to 6, with the total score summation ranging from 0 to 39 for each substance. Total scores between 0 and 3 (0–10 for alcohol) are considered low risk (occasional or non-harmful use), 4 and 26 (11–26 for alcohol) indicate moderate risk (more regular use or harmful/hazardous use), and scores higher than 26 indicate high risk (frequent high-risk use or suggestive of dependence)	Cronbach's alpha values ranging from .71 to .90.

**P4 Screener**	4	If a positive answer is given to the pre-screening question about thoughts of self-harming, there are subsequent questions on: Past suicide attempts, Plan, Probability of completing suicide, and Preventive factors.	*Minimal risk*= no past history, no suicide plan, and no probability of an attempt. *Lower risk*= a plan and/or past history, but no probability of an attempt; presence of preventive factors. *Higher risk*= probability of a suicide attempt; absence of preventive factors.	NA
**Academic Motivation Scale (AMS)**	20	11-point Likert scale from 0 (Not at all true) to 10 (Completely true).	5 sub-scales Each sub-scale score ranges from 0 to 40 (no cut-off).	Cronbach's alpha values ranging from .73 to .91
**Perceived School Self-Efficacy Scale (PSSES)**	9	5-point Likert scale from 1 (Not capable at all) to 5 (Fully capable).	The total score ranges from 9 to 45 (no cut-off).	Cronbach's alpha values ranging from .83 to .87.
**Self-Regulated Knowledge Scale-University (SRKS-U)**	15	5-point Likert scale from 1 (Never) to 5 (Always or nearly always).	5 sub-scales Each sub-scale score ranges from 3 to 15 (no cut-off).	Cronbach's alpha values ranging from .70 to .80
**Shortened Achievement Emotion Questionnaire (AEQ-S)**	32 item for the sub-scale "Learning-related Emotions"	5-point Likert scale from 1 (Strongly disagree) to 5 (Strongly agree).	The sub-scale is computed by summing the items and taking the mean (no cut-off).	Cronbach's alpha values ranging from .75 to .93.
**South Oaks Gambling Screen (SOGS)**	20	The response options for items are dichotomous (Yes or No).	Scores on the SOGS are determined by scoring one point for each question that shows the "at risk" response indicated and adding the total points. The maximum score is 20 (cut-off ≥ 5)	Cronbach's alpha of .97.

Note. NA: Not Applicable.

Table S2. Descriptive statistics and comparison of the socio-demographic and clinical features between the two survey samples.

	Survey 2019 n=553	Survey 2022 n=721	P value
**Gender**			
Male	234 (42.3%)	260 (36.1%)	.070

Female	317 (57.3%)	457 (63.4%)	
Other	2 (0.4%)	4 (0.6%)	
**Age**			
Mean (SD)	22.9 (3.80)	20.8 (3.83)	<.001 (t)
**Nationality**			
Italian	527 (95.3%)	685 (95.0%)	.914
Other	26 (4.7%)	36 (5.0%)	
**Marital status**			
Single	257 (46.5%)	407 (56.4%)	<.001
Relationship	296 (53.5%)	314 (43.6%)	
**University status**			
Student	424 (76.7%)	473 (65.6%)	<.001
Worker	129 (23.3%)	248 (34.4%)	
**Living status**			
In town	344 (62.2%)	517 (71.7%)	<.001
Out-town	209 (37.8%)	204 (28.3%)	
**UCS Total**			
Mean (SD)	81.4 (17.6)	83.4 (17.1)	.073
**GHQ-12 Total**			
Mean (SD)	6.44 (3.00)	6.07 (2.92)	.029
**P4 Screener (suicide risk)**			
No	463 (83.7%)	610 (84.6%)	.727
Yes	90 (16.3%)	111 (15.4%)	
**USS Total**			
Mean (SD)	14.3 (7.54)	14.4 (8.39)	.783
**Brief COPE Active coping**			
Mean (SD)	5.29 (1.39)	5.58 (1.40)	<.001
**Brief COPE Planning**			
Mean (SD)	5.97 (1.47)	5.64 (1.54)	<.001
**Brief COPE Positive reframing**			
Mean (SD)	4.81 (1.61)	4.76 (1.55)	.722
**Brief COPE Acceptance**			
Mean (SD)	5.60 (1.42)	5.44 (1.48)	.035
**Brief COPE Humor**			
Mean (SD)	3.82 (1.54)	3.97 (1.52)	.0396
**Brief COPE Religion**			
Mean (SD)	3.03 (1.56)	3.01 (1.55)	.786

**Brief COPE Emotional support**			
Mean (SD)	4.66 (1.71)	4.62 (1.71)	.765
**Brief COPE Instrumental support**			
Mean (SD)	4.78 (1.64)	4.73 (1.68)	.488
**Brief COPE Self-distraction**			
Mean (SD)	5.07 (1.46)	5.03 (1.43)	.747
**Brief COPE Denial**			
Mean (SD)	2.75 (1.13)	2.83 (1.17)	.291
**Brief COPE Venting**			
Mean (SD)	4.48 (1.61)	4.37 (1.54)	.343
**Brief COPE Substance Use**			
Mean (SD)	2.37 (1.01)	2.46 (0.999)	.003
**Brief COPE Behavioural disengagement**			
Mean (SD)	3.12 (1.28)	3.29 (1.41)	.069
**Brief COPE Self-blame**			
Mean (SD)	5.84 (1.49)	5.69 (1.56)	.153
**ASSIST Tobacco**			
Mean (SD)	5.16 (7.28)	3.81 (7.21)	<.001
**ASSIST Alcohol**			
Mean (SD)	7.11 (5.97)	4.09 (4.16)	<.001
**ASSIST Marijuana**			
Mean (SD)	1.76 (4.61)	0.638 (2.76)	<.001
**ASSIST Cocaine**			
Mean (SD)	0.0850 (0.831)	0.0166 (0.380)	.019
**ASSIST Stimulants**			
Mean (SD)	0.0325 (0.360)	0.0319 (0.587)	.276
**ASSIST Inhalants**			
Mean (SD)	0.0217 (0.312)	0.0291 (0.583)	.745
**ASSIST Sedatives**			
Mean (SD)	0.544 (3.07)	0.341 (2.21)	.050
**ASSIST Hallucinogens**			
Mean (SD)	0.0488 (0.458)	0.0291 (0.538)	.089
**ASSIST Opioids**			
Mean (SD)	0.0452 (0.876)	0 (0)	.106
**ASSIST Other drugs**			
Mean (SD)	0.0217 (0.312)	0.0139 (0.247)	.743

Table S3. Distribution of responses to the three main outcomes regarding the effect of pandemic on social relationship and academic life (survey 2022).

Outcome categorical variables	n (%)
**Pandemic impact on social relationship** [yes *vs* no]	
Yes	429 (78%)
No	124 (22%)
**Pandemic impact on academic grade: pandemic improves academic grade** [yes *vs* no]	
Yes	222 (40%)
No	331 (60%)
**Pandemic impact on academic performances: pandemic worsened academic performances** [yes *vs* no]	
Yes	260 (47%)
No	293 (53%)
